# On the Sensitivity of the Parameters of the Intensity-Based Stochastic Model for Terrestrial Laser Scanner. Case Study: B-Spline Approximation

**DOI:** 10.3390/s18092964

**Published:** 2018-09-05

**Authors:** Gaël Kermarrec, Hamza Alkhatib, Ingo Neumann

**Affiliations:** Geodetic Institute, Leibniz Universität Hannover, Nienburger Str. 1, 30167 Hannover, Germany; alkhatib@gih.uni-hannover.de (H.A.); neumann@gih.uni-hannover.de (I.N.)

**Keywords:** terrestrial laser scanner, stochastic model, B-spline approximation, control point, intensity-based model

## Abstract

For a trustworthy least-squares (LS) solution, a good description of the stochastic properties of the measurements is indispensable. For a terrestrial laser scanner (TLS), the range variance can be described by a power law function with respect to the intensity of the reflected signal. The power and scaling factors depend on the laser scanner under consideration, and could be accurately determined by means of calibrations in 1d mode or residual analysis of LS adjustment. However, such procedures complicate significantly the use of empirical intensity models (IM). The extent to which a point-wise weighting is suitable when the derived variance covariance matrix (VCM) is further used in a LS adjustment remains moreover questionable. Thanks to closed loop simulations, where both the true geometry and stochastic model are under control, we investigate how variations of the parameters of the IM affect the results of a LS adjustment. As a case study, we consider the determination of the Cartesian coordinates of the control points (CP) from a B-splines curve. We show that a constant variance can be assessed to all the points of an object having homogeneous properties, without affecting the a posteriori variance factor or the loss of efficiency of the LS solution. The results from a real case scenario highlight that the conclusions of the simulations stay valid even for more challenging geometries. A procedure to determine the range variance is proposed to simplify the computation of the VCM.

## 1. Introduction

Observations from a terrestrial laser scanner (TLS) are inherently noisy measurements, i.e., range as well as angles are influenced by random effects and are thus stochastic quantities. Instrumental errors such as scale error, zero point error, or collimation axis error can be taken into account thanks to manufacturer’s descriptions, see e.g., Boehler and Marbs [1], and Litchi et al. [2]. Because these specifications may change after using the instrument for a long time, Li et al. [3] developed an innovative self-calibration method. They further highlighted that the mirror tilt and the vertical index offset errors are the major error sources and should be corrected to improve the positional accuracy of point clouds. Besides instrumental errors, random effects influence the precision of TLS range measurements. The incidence angle was shown empirically to be one of the major influencing factor [4,5,6], besides the range itself [7], or the sampling rate used [8]. Furthermore, Zamecnikova et al. [9] highlighted that the color and roughness of the target affect the variance of range measurements. The impact of temperature and pressure should not be ignored by using an elementary model [10]. Further atmospheric random effects coming from the propagation through a turbulent medium [11] are, however, more challenging to model. The induced correlations remain thus mostly omitted although empirical studies highlighted strong spatial correlations under particular scanning geometries [12]. Therefore, only simulations were up to now used to assess the impact of physical correlations when TLS observations are used in a least-squares (LS) adjustment [13].

To account for the heteroscedasticity of range measurements, different models were derived by Ozendi et al. [6], Wujanz et al. [14,15] and Lambertus et al. [5]. All proposals make use of intensity values, i.e., the optical power of the backscattered echo of the emitted signal. The underlying assumption is thus simple: a range measurement corresponding to low intensity values is less trustworthy and should be downweighted accordingly. This idea is physically plausible [16,17] and was widely used in other domains. For example, the observations of GPS measurements can be weighted by means of signal to noise ratio (SNR) values [18,19].

Besides a theoretical basis, the main advantage of these so-called TLS intensity models (IM) is their ability to resume many of the previously described random effects that were empirically shown to influence the range variance. Because the intensity physically depends on the scanning geometry, the properties of the reflected target and to some extent the travelled medium, these proposals have, with reason, gained attention. The simplest model was proposed by Wujanz et al. [14], who fitted the variance versus raw intensity values with a power function involving two parameters: a scaling and a power factor. For other laser scanners, the addition of a constant was necessary, i.e., a white noise contribution [15]. Focusing on the range variance for low intensities for which a correct weighting is of greater significance, different power factors were empirically found [20]. Unfortunately, the drawback of all IM is the necessity to determine the corresponding model parameters by means of time-demanding methods, from calibration in 1D mode to the most common residual analysis of LS adjustment. Additionally, a user may doubt if the parameters estimated under optimal conditions are still valid for its own application.

As TLS measurements are increasingly used for deformation analysis, a post processing in LS adjustment is necessary to determine e.g., the parameters of a plane with a Gauss-Helmert Model (GHM) [21], or the control points (CP) in B-splines curve or surface approximation [22]. For a trustworthy LS solution, the aforementioned heteroscedasticity of range measurements should be taken into account and modeled adequately. Indeed, both the volume and orientation of the error ellipsoid are impacted by the parameters of an estimated variance model [23], as well as the a posteriori variance factor. Thus, misspecifications of the stochastic model propagate in the LS solution, making results of the overall model test (also called global test) or outlier test less interpretable [24,25].

The aforementioned point-wise intensity variance model is a first answer to describe more accurately the randomness of TLS range measurements and to enhance the trustworthiness of the derived LS solution. The need for further refinements to fix the parameters of the IM is however questionable, particularly when the observations are post processed in a LS adjustment. In this contribution, we investigate how the parameters of the point-wise IM affect the loss of efficiency of the LS solution and the a posteriori variance factor. These carefully chosen quantities are central to judge the trustworthiness of the LS adjustment. Because they are impacted by the misspecifications of the IM parameters and can be evaluated by means of closed formulas, they were previously used by Kermarrec and Schön [23] for the sensitivity analysis of a parametric correlation model. We thus analyze how variations of the IM parameters affect these quantities in order to determine in which cases a simplification of the stochastic model would be relevant. We make use of simulations for which the VCM of the observations can be exactly fixed and the geometry of the problem perfectly controlled. Because of its flexibility and potential for deformation analysis [26], we focus on the determination of the CP of B-splines curve approximation [27,28]. The presented results can be easily extended to surface fitting or for the determination of geometric primitives with GHM.

The remainder of this paper is structured as follows: the first section provides a brief summary of the mathematical concepts of least-squares and stochastic modeling, introducing the intensity-based model for the range variance. Two quantities are defined: the a posteriori variance factor and the loss of efficiency, which allow quantifying the impact of misspecification of the parametric stochastic model. The third section describes the results of simulations for a geometry free case whereas the fourth section provides a description of a real case study. A proposal to fix the constant variance concludes the contribution.

## 2. Mathematical Background

### 2.1. Functional Model 

Free-form curve and surface fittings are flexible tools to approximate point clouds [22] without being restricted by the use of geometric primitives, such as circle, planes, or cylinders. In this contribution, we make use of B-splines. Their properties and advantages over other functions, such as control and flexibility, are exemplarily described in Bureick et al. [27]. As B-splines also allow for surface fitting, they gain an increasing interest in the field of engineering geodesy. Readers interested in more details on how spline fitting works should refer e.g., to de Boor [22], Piegl and Tiller [29], and more specifically for geodetic applications to Koch [30], and Bureick et al. [27]. For the sake of shortness, we shortly introduced the main concept, focusing on the stage where the stochastic model of the observations is involved, i.e., the determination of the CP by LS adjustment.

Control points define a rough sketch of the curve: the control polygon of the spline. A B-spline curve over a given knot vector is thus completely determined by its CP, which change locally the shape of the approximation. The distance between the curve and the observations has to be minimized in the mean square sense, in the metric of the observations. Thus, after the parameterization of the curve and the determination of the knot vector, the coordinates of the control points (CP) are usually determined by LS adjustment. The number of CP to be determined is often left to the user, provided that the order of the spline does not exceed the number of CP plus 1. Information criterion such as BIC, AIC [31] or structural risk minimization [28] can provide useful information about the optimal number of CP to choose. For the sake of smoothness, B-splines of third order are preferred, i.e., the degree chosen determines the basis function of the decomposition by means of a recursive formula [22]. For particular cases, such as the determination of a simple line, an order 0 is more relevant, for which the corresponding B-spline takes the value 1 between two interval knots and 0 outside.

In this contribution, we place ourselves in the position of a TLS user wanting to account for heteroscedasticity of the TLS observations for post processing analysis. We focus on the intensity-based stochastic model as proposed in Wujanz et al. [14,15]. This proposal has the main advantage of involving only few parameters, by having shown empirically to be accurate to describe the variance of the range measurements. Because different power and scaling factors were reported in the literature for various TLS, we study here the impact of varying the parameters of the IM, when used in the LS adjustment to determine the CP of the B-splines approximation.

The estimated quantities of the adjustment are the three Cartesian coordinates of the CP. We call *u* the number of CP. The observation equation in matrix form reads:

(1) l=Ax+v,

where the observation matrix is $l=\left[l_{x}\;l_{y}\;l_{z}\right]$ and corresponds to the n×3 Cartesian coordinates of the point clouds. x is the u×3 parameter vector to be estimated and v is the n×3 vector of the random errors. We let $E\left(v\right)=0,\;E\left(vv^{T}\right)=\sigma_{0}^{2}Q_{0,MC}$, $\sigma_{0}^{2}$ is the a priori variance factor and $Q_{0,MC}$ the true VCM of the observations, the subscript *MC* meaning mathematical correlation. E(·) denotes the mathematical expectation. The normality of the residuals is a necessary condition for the validity of test statistics such as the global or outlier tests [32].

The design matrix describes the relationship between the estimates and the observations. It is built block wise as follows:

(2) $$A=\left[\begin{array}{ccc} A_{CP} & 0 & 0\\ 0 & A_{CP} & 0\\ 0 & 0 & A_{CP} \end{array}\right]$$

The elements of $A_{CP}$ of size (n,u) are derived from the values of the B-splines basis functions [27].

The LS estimator of Equation (1) is given by:

(3) $${\hat{x}}_{0}={\left(A^{T}Q_{0,MC}^{-1}A\right)}^{-1}A^{T}Q_{0,MC}^{-1}l$$

We have $E\left({\hat{x}}_{0}\right)=x_{0}$, $x_{0}$ being the true and unknown solution. The a posteriori variance factor of the observations ${\hat{\sigma }}_{0}^{2}$ is expressed as:

(4) $${\hat{\sigma }}_{0}^{2}=\frac{{\left(l-A{\hat{x}}_{0}\right)}^{T}Q_{0,MC}^{-1}\left(l-A{\hat{x}}_{0}\right)}{n-3u}=\frac{{\hat{v}}_{0}^{T}Q_{0,MC}^{-1}{\hat{v}}_{0}}{n-3u},$$

with $E\left({\hat{\sigma }}_{0}^{2}\right)=\sigma_{0}^{2}$ [24,33].

The cofactor matrix $Q_{0,\hat{x}\hat{x}}$ of the unknows (a priori estimator) is given by:

(5) $$Q_{0,\hat{x}\hat{x}}={\left(A^{T}Q_{0,MC}^{-1}A\right)}^{-1}$$

### 2.2. Stochastic Model for TLS

TLS measurements are made of two angles, horizontal and vertical (HA and VA respectively) and a range, also called distance. The stochastic modelling of the range is the focus of this contribution. No physical correlation are considered, as up to now no satisfactory description exists. In the following, we assume a constant standard deviation of 7.7 mgon for both normally distributed VA and HA [34]. 

#### 2.2.1. Stochastic Model for Range Measurements

Based on calibrations using the 1D mode of TLS or residual analysis, the standard deviation of the range versus intensity was fitted for different materials and sampling rate with a power function [14]:

(6) $$\sigma_{r}^{}=c+\beta Int^{\alpha }$$

The parameters α, β and c were determined empirically by regression analysis for different laser scanners so that no direct relationship with a physical model based on noise description was searched. c models a constant white noise added to an intensity based variance model. It has no impact on the shape of $\sigma_{r}^{}$ when Int is varied. The variance of the range is $\sigma_{r}^{2}$.

For the first phase TLS under consideration (Z + F Imager 5006 h), α was found to be close to −12 for all sampling rates [14]. This value is coherent with the physical derivation of SNR based variance model [16], intensities and SNR being equivalent. Further contributions [15] found for phase TLS empirically higher α close to −0.8. The range of values of β was shown to depend on the sampling rate of the measurements and was reported to vary from approximately 1 to 11 mm, i.e., an expected decreasing precision of the range for lower sampling. A slightly different set of parameters [α,β,c] were found for time of flight laser [15] or for intensities below 10^4^ [20].

**Figure 1:**
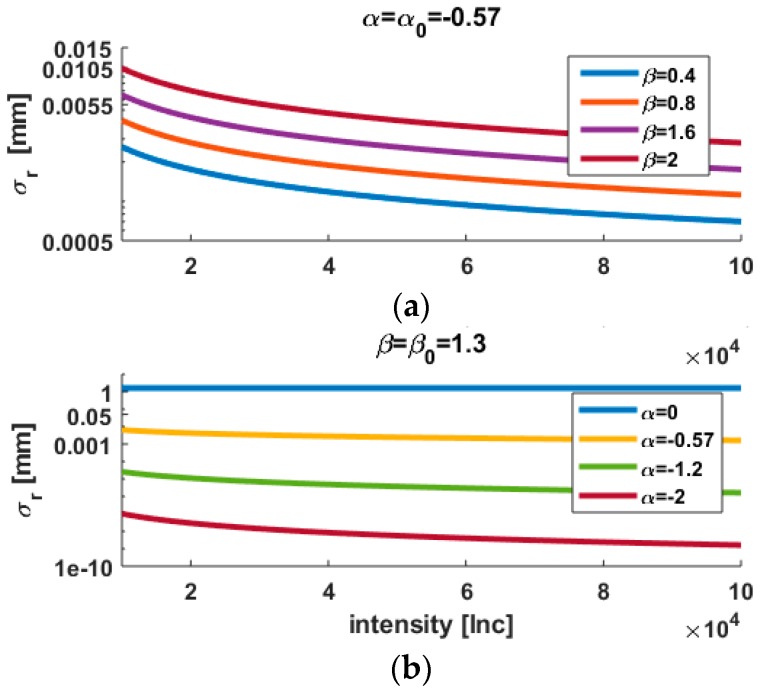
Variations of $\sigma_{r}$ (log plot) versus intensity with the intensity-based model (**a**) for different the scaling factors β and (**b**) different power factors α.

In this contribution, we will consider without loss of generality that c=0. This additional parameter models a white noise contribution. Figure 1 presents the variations of $\sigma_{r}^{}$ versus Int. The raw intensity values were taken in a range from 10^4^ to 10^5^ and are expressed in increment (Inc). Whereas the upper panel corresponds to variations of β for a fix α, in the lower one α is varied and β kept constant to 1.3. This value corresponds to a sampling rate of 1016 kHz in Wujanz et al. [14]. Unsurprisingly, β acts as a rescaling parameter of the variance. When α is decreased however, the variations of the standard deviation are more pronounced at low intensities. $\sigma_{r}^{}$ approaches a constant value when α tends to 0.

#### 2.2.2. Note on Scaling

When for a given intensity, two models corresponding to different set of parameters [α,β,c] give the same standard deviation, they are called “scaled models”. This property is of main importance when comparing parametric models with each other, as it defines the metric. Exemplarily, increasing c by keeping β constant in Equation (6) corresponds to decreasing the influence of the intensity dependency on the total variance, provided that the models are scaled. β and c are thus linked with each other. Mathematically, the scaling is summarized as follows: for a given reference intensity $Int_{ref}$, the reference standard deviation $\sigma_{r,ref}$ is expressed following Equation (6), i.e., $\sigma_{r,ref}=c_{ref}+\beta_{ref}Int_{ref}^{\alpha_{ref}}$, where $\left[\alpha_{ref},\beta_{ref},c_{ref}\right]$ is a given reference set. A model is said to be scaled with the reference if $\forall c,\beta ,\alpha \;\sigma_{r}^{s}\left(Int_{ref}\right)=\sigma_{r,ref}$, where $\sigma_{r}^{s}$ is the scaled standard deviation.

#### 2.2.3. Building the VCM of the TLS Measurements

An approximated diagonal variance cofactor matrix $\hat{\Sigma }$ of size (n,n) models the main uncertainties of the TLS measurements, i.e., range, horizontal and vertical angles. The block matrices ${\hat{\Sigma }}_{r},{\hat{\Sigma }}_{HA},{\hat{\Sigma }}_{VA}$ are sorted point-wise and the diagonal *i*th element of each submatrix is given by $q_{r}(i,i)=\sigma_{r(i)}^{2},q_{HA}(i,i)=\sigma_{HA(i)}^{2},q_{VA}(i,i)=\sigma_{VA(i)}^{2}$ and

(7) $$\hat{\Sigma }=\left[\begin{array}{ccc} {\hat{\Sigma }}_{r} & 0 & 0\\ 0 & {\hat{\Sigma }}_{HA} & 0\\ 0 & 0 & {\hat{\Sigma }}_{VA} \end{array}\right]=\sigma_{0}^{2}\hat{Q},$$

where $\sigma_{0}^{2}$ is the a priori variance factor and $\hat{Q}$ the cofactor matrix of the observations.

#### 2.2.4. Mathematical Correlations

The result of the final LS adjustment after having parametrized the curve and determined the knot vector are the Cartesian coordinate of the CP. The original observations from the TLS (i.e., range and angles) have thus to be transformed from polar to Cartesian coordinates. We use the error propagation law to compute the VCM ${\hat{Q}}_{MC}$ of the Cartesian coordinates of the point cloud, where the subscript *MC* means mathematical correlations:

(8) $${\hat{Q}}_{MC}=F\hat{Q}F^{T}$$

The matrix F is the Jacobian matrix containing the derivatives of the point coordinates with respect to the range and angles. For one point *i*, $F_{i}$ reads:

$$F_{i}=\left[\begin{array}{ccc} \sin(VA_{i})\cos(HA_{i}) & r_{i}\cos(VA_{i})\cos(HA_{i}) & -r_{i}\sin(VA_{i})\sin(HA_{i})\\ \sin(VA_{i})\sin(HA_{i}) & r_{i}\cos(VA_{i})\sin(HA_{i}) & r_{i}\sin(VA_{i})\cos(HA_{i})\\ \cos(VA_{i}) & -r_{i}\sin(VA_{i}) & 0 \end{array}\right].$$

The result of the transformation is a sparse fully populated VCM ${\hat{Q}}_{MC}$ with a block structure.

### 2.3. Effect of Misspecification of the VCM

Unfortunately only the so-called feasible weighted LS [35] is assessable and $Q_{0,MC}$ in Equation (3) is replaced by its estimates ${\hat{Q}}_{MC}$. We call the FWLS estimator:

(9) $$\hat{x}={\left(A^{T}{\hat{Q}}_{MC}^{-1}A\right)}^{-1}A^{T}{\hat{Q}}_{MC}^{-1}l.$$

and

(10) $${\hat{\sigma }}_{\hat{Q}}^{2}=\frac{{\left(l-A\hat{x}\right)}^{T}{\hat{Q}}_{MC}^{-1}\left(l-A\hat{x}\right)}{n-3u}=\frac{{\hat{v}}^{T}{\hat{Q}}_{MC}^{-1}\hat{v}}{n-3u}.$$

When the computation of ${\hat{Q}}_{MC}$ is based on a parametric model, misspecifications of the parameters propagate in the FWLS solution, i.e., the obtained estimator is not trustworthy. Its precision may be over- or underoptimistic with respect to the truth, as seen in Equation (3) [23]. As long as the stochastic model is not strongly misspecified and enough observations are taken into account, the LS estimator is unbiased so that $\forall \hat{Q},\;E\left(\hat{x}\right)=x_{0}$. As a consequence, the chosen VCM has a low impact on the parameters to be estimated [36]. On the contrary, quantities such as the a posteriori variance factor, shown in Equation (4), or the cofactor matrix, seen in Equation (5), are affected by changes of the VCM. This property is particularly visible when different scaling is used, i.e., the inverse of the VCM is involved only once so that e.g., a factor in ${\hat{Q}}_{MC}$ changes Equations (4) and (5) in the same proportion.

Following the work of Kermarrec and Schön [37], who studied the impact of varying the parameters of a correlation function for GPS phase measurements, or Jurek et al. [13] for TLS observations, we will analyse how variations of α and β in $\hat{Q}$ influence the LS solution. Two quantities are chosen to study the impact of model misspecification:

- The a posteriori variance factor, which is a key quantity in many statistical tests [2] such as the overall model test, and allows judgment of the LS solution.
- The loss of efficiency of the estimator, which is based on a ratio of mean-squared errors (MSE). It measures the performance of the LS estimator: an MSE close to 0 means that the estimator has a perfect accuracy. MSE should only be used for comparative purposes. The main advantage of the MSE formulation is its nondependency on the dataset allowing still a quantification of the mean-squared differences of the estimated parameters when VCM are changed [37].

For these two quantities, the bias due to approximated VCM is available by means of closed formula, i.e., no Monte Carlo simulations are necessary.

Up to now, we let ${\hat{Q}}_{MC}=Q_{0,MC}+\Delta Q$ represents the a priori matrix, where ΔQ is the difference between the assumed ${\hat{Q}}_{MC}$ and the true cofactor matrix $Q_{0,MC}$. The expected bias of the variance of unit weight due to the incorrect weights on the estimate can be expressed [25] as:

(11) $$E\left({\hat{\sigma }}_{Q}^{2}\right)=\sigma_{0}^{2}+\mathrm{tr}\left\{\left(I-{\hat{Q}}_{MC}^{-1}A{\left(A^{T}{\hat{Q}}_{MC}^{-1}A\right)}^{-1}A^{T}\right){\hat{Q}}_{MC}^{-1}\Delta Q\right\}\frac{\sigma_{0}^{2}}{n-u}=\sigma_{0}^{2}\left(1+BIAS\right),$$

where tr denotes the trace of a matrix, and I the identity matrix. BIAS depends both on ${\hat{Q}}_{MC}$ and ΔQ and can be either positive or negative.

The loss of efficiency in estimating $\hat{x}$ instead of ${\hat{x}}_{0}$ is given by the nonnegative definite matrix [24].

$$Q_{\hat{x}\hat{x}}-Q_{0,\hat{x}\hat{x}}={\left(A^{T}{\hat{Q}}_{MC}^{-1}A\right)}^{-1}A^{T}{\hat{Q}}_{MC}^{-1}Q_{0,MC}{\hat{Q}}_{MC}^{-1}A{\left(A^{T}{\hat{Q}}_{MC}^{-1}A\right)}^{-1}-{\left(A^{T}Q_{0,MC}^{-1}A\right)}^{-1}$$

The quantity:

(12) $$R_{\mathrm{MSE}}=\frac{\mathrm{tr}\left(\left[{\hat{Q}}_{MC}^{-1}A{\left(A^{T}{\hat{Q}}_{MC}^{-1}A\right)}^{-2}A^{T}{\hat{Q}}_{MC}^{-1}\right]Q_{0,MC}^{}\right)}{\mathrm{tr}\left({\left(A^{T}Q_{0,MC}^{-1}A\right)}^{-1}\right)}-1,$$

was proposed in Kermarrec and Schön [37] or Stein [38] to analyse the influence of changes in the structure of the estimated covariance matrices on the a priori precision, a minimum of the ratio being searched.

## 3. Simulation

### 3.1. Methodology

Because Equations (3) and (5) depend both on the true but in real cases unknown VCM $Q_{0,MC}$, simulations remain the only way to assess the impact of an approximated VCM ${\hat{Q}}_{MC}$ on the quantities of interest. In that case, $Q_{0,MC}$ is fixed following Equation (8). The reference values for the IM are taken from Wujanz et al. [14] to ${\left[\alpha ,\beta \right]}_{0}=[-0.57,1.6]$.

The chosen methodology for the simulations relies on the computation of observed-minus-computed (OMC) observations, similarly to what is done for GPS positioning. In a first step, we consider starting from TLS observations corresponding to a given intensity vector and aim thus to eliminate the geometry from the TLS measurements. This way, the VCM of the OMC TLS observations is fully under control. We summarize the corresponding steps as follows:

- The Cartesian observations l are approximated with a B-splines curve by assuming that ${\hat{Q}}_{MC}=I$. I is the identity matrix. i.e., no assumption about the stochastic properties of the TLS measurements is made in this first step. The design matrix A is filled using the knot vector and B-splines of order 3 [27], as seen in Equation (2).
- The computed observations are obtained from $l_{computed}=A\hat{x}$, where $\hat{x}$ are the coordinates of the estimated CP.
- The OMC observations are given by $l-l_{computed}$. They thus correspond to having extracted the geometry from the original observations: $l-l_{computed}$ is a zero-mean vector (or a straight line). The VCM of the OMC measurements corresponds exactly to $Q_{0,MC}$ but will be replaced by its approximation ${\hat{Q}}_{MC}$ in the second LS adjustment, i.e., the approximation of the straight line. In this last step, the determination of the knot vector and by extension of the design matrix are straightforward and we choose an order 0 for the B-splines. A is filled up with 1, and only one control point is estimated, which corresponds to the mean estimator.

By considering a “geometry free” case, we intentionally focus only on analysing the impact of an approximated stochastic model on the adjustment, i.e., no functional model misspecification has to be considered. Clearly, this corresponds to straight-line observations having known stochastic properties. The described procedure is a way to explain how such a situation can be built from original TLS observations. In order to generalize our conclusions, a case study corresponding to a less simple design matrix will be presented in Section 4.

### 3.2. Intensity Vector

**Figure 2:**
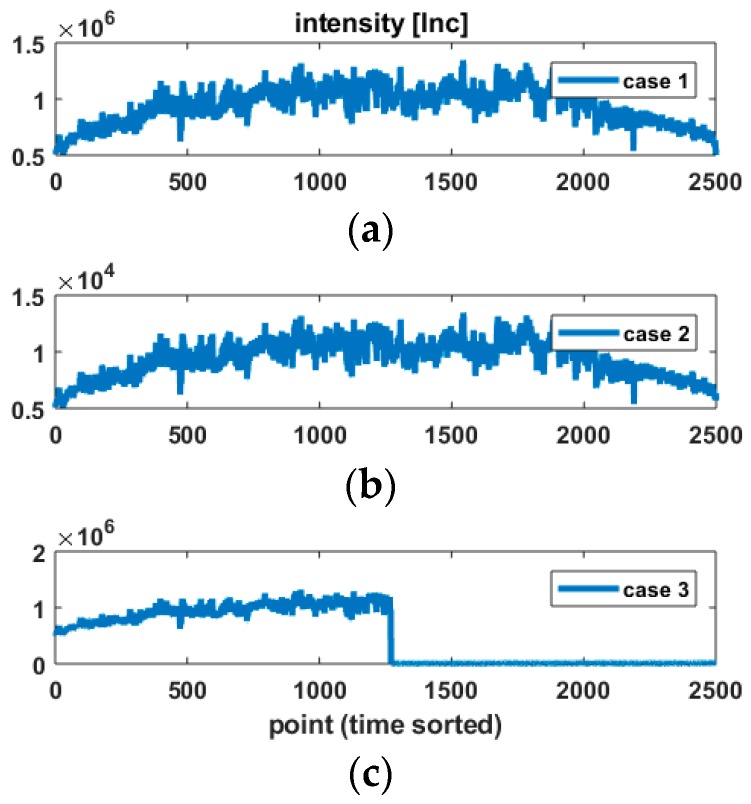
The three intensity vectors retained for the simulations. The intensity values in [Inc] are sorted per point, i.e., per time. 2500 points were measured. Intensity vector (**a**) $Int_{ref}$ corresponds to an original vector from an arch bridge; (**b**) $Int_{ref}/100$; (**c**) a two part intensity vector built as a mix between case 1 and case 2.

Equation (6) takes for granted that the intensity is known and saved together with the TLS measurements. In order to analyse the impact of varying the parameters [α,β], a plausible intensity vector $Int_{ref}$, which contains the intensity values sorted temporally, corresponding to the considered real case. Figure 2a presents the chosen reference intensity vector. As a 2D profile mode was used, it is sorted temporally, one profile corresponding to variations of the VA. The object under investigation was an arch bridge, explaining thus the symmetry of the original vector. A detailed description of the bridge structure and experiment can be found in Schacht et al. [39]. 

The variance of the range measurements depends on the intensity, as seen in Figure 1. Thus, in order to simulate different cases that occur in real data analysis, three different VCM $Q_{0,MC}$ were computed. They correspond to different intensity vectors Int that are derived from $Int_{ref}$:

- Case 1: the intensity vector is the original one $Int_{ref}$, seen in Figure 2a. The mean intensity value is 930,000 Inc, which corresponds to a mean estimated standard deviation for the range of $\sigma_{r}=0.64\mathrm{mm}$.
- Case 2: the intensity is the same as case 1, rescaled by a factor 1/100. By doing so, we aim to simulate low intensity values, seen in Figure 2b. Following Figure 1, a higher variability of the range variance because of the power function is expected. A higher impact on the a posteriori variance factor or the loss of efficiency when α or β are misspecified can be expected.
- Case 3: the intensity profile corresponding to case 1 is divided in the middle into two parts, seen in Figure 2c: the first part corresponds to case 1, whereas the second one to case 2. The intent here is to simulate intensity values that would vary strongly inside the same object due e.g., to different reflectivity properties.

### 3.3. Scaling of $\sigma_{r}$

When the parameter β and α are varied, the values of the standard deviation $\sigma_{r}$ for a given intensity value will change accordingly as shown in Figure 1. As mentioned previously, β acts as a scaling parameter for the variance. Thus, comparisons between different VCM are still possible yet meaningless as the metrics of the VCMs are different: the scaling factor will influence Equation (11) through ΔQ. In order to study the impact of the variations of the power factor α only, the influence of different metrics should be reduced. We propose thus to scale $Q_{0}$ and $\hat{Q}$ as follows:

(13) $$\sigma_{r,s}=\sigma_{r}\frac{\left(\beta_{0}{\left(\bar{I}nt\right)}^{\alpha_{0}}\right)}{\left(\beta {\left(\bar{I}nt\right)}^{\alpha }\right)},$$

where $\bar{I}nt$ is the mean of the intensity vector, and $\sigma_{r,s}$ states for the scaled standard deviation of the range. Intentionally, the mean value of Int has been retained instead of its maximum or minimum value. This strategy temps to counterbalance the possible effects of outliers that may be present in the intensity, i.e., high or low values that are not relevant. This proposal refers to the work of Luo et al. [18] for SNR weighting applied to GPS observations.

### 3.4. Results of Simulations

#### 3.4.1. Varying α

Physical considerations would allow the fixing of α to −0.5 [11]. However, empirical determinations based on calibrations have shown that values of −0.8 were possible for phase TLS. We propose to vary α in the interval $\left[\begin{array}{cc} -0.9, & 0 \end{array}\right]$. The case α=0 corresponds to a constant $\sigma_{r}$ and is thus independent of the underlying variation of the intensity, i.e., the VCM is just a scaled identity matrix. To the point of view of the authors, taking α=−0.9 is an exaggerated assumption when c is taken to 0. The impact of higher values can be easily extrapolated from the obtained results. Values of α<−0.5 are similar to assuming a stronger impact of the intensity on the total variance for scaled models, which can be counterbalanced by the introduction of the parameter c, as seen in Equation (6).

**Figure 3:**
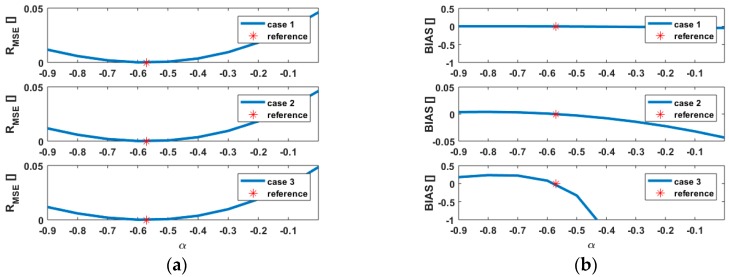
Impact of variations of the power factor α of the intensity-based model for R_MSE_ (**a**) and BIAS (**b**); the three subplots correspond to the three cases of interest: normal (1), low (2) and strong varying intensities (3) respectively. Please note the different scaling for case 3 (bottom b) for the sake of readability.

The quantities defined in Equations (11) and (12) are plotted versus α and presented in Figure 3 for the three scenarios. Please note that the same shape may however correspond to different cases, e.g., 1 and 2.

- Case 1 and 2

From Figure 3a (top and middle), the quantities of interest are not impacted by variations of α and remain similar for both case 1 and 2, although the variations of $\sigma_{r}$ were shown to be stronger for low intensities, seen in Figure 1a. The variations of R_MSE_ follow the standard symmetric shape of a mean squared error around 0 so that R_MSE_ is slightly lower for α=−0.9 than for α=0, the difference of 0.04 (4%) being considered as non-relevant. The case α=0 retains our attention since it corresponds to a simplification of the weighting model, which becomes independent of the intensity. The value BIAS reaches in that case a negligible value of −0.046. Although it corresponds to a decrease of 9% wrt. to the 0-reference, the value in itself is not significant, i.e., an overall test [40] will not be sensitive to such a variation. Thus, a misspecification of α under the same metric impacts neither BIAS nor R_MSE_ significantly in the first two cases.

- Case 3

The case 3 corresponds to strong variations of the intensity values. Figure 3a (bottom) highlights that the variations of BIAS are much pronounced than in the first two cases. Indeed, for α=−0.9, BIAS=0.2, which is more than 40 times higher than in case 1 and 2. Clearly, less variations around the true parameter $\alpha =\alpha_{0}$ are allowed to stay in the previous range of values for BIAS, e.g., in our case when $\alpha_{0}-0.04<\alpha <\alpha_{0}+0.02$, |BIAS|<0.05. Because of the rate of change of BIAS, the upper bound of α has to be tightened compared to the lower bound as seen in Figure 3a (bottom). From α=−0.2, BIAS decreases to incoherent negative values. Indeed, from Equation (11), 1+BIAS should always be positive, i.e., $E\left({\hat{\sigma }}_{\hat{Q}}^{2}\right)>0$. However, for the range of values α∈[−0.2,0], BIAS<−1 does not fulfil this condition since ΔQ cannot be considered as small anymore, e.g., a value of ${\left\|\Delta Q\right\|}_{2}\approx 16,000$ for α=0 was found, ${\left\|\cdot \right\|}_{2}$ being the L2 norm. In that particular case, the unbiased nature of the LS estimator is furthermore questionable. The stochastic model can be considered as strongly misspecified, which will negatively influence the estimated CP coordinates.

#### 3.4.2. Varying β

**Figure 4:**
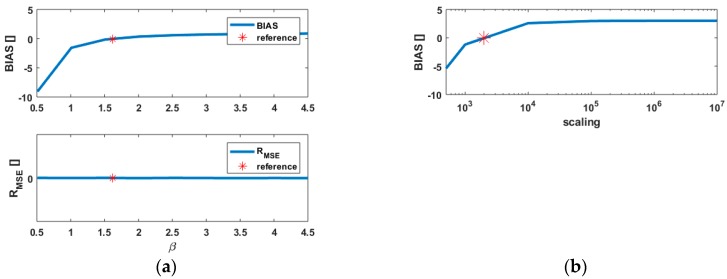
(**a**) Impact of variations of the scaling β of the intensity-based model for the bias of the loss of efficiency R_MSE_ (**top**) and the a posteriori variance factor (**bottom**). All cases of interest lead to the same values; (**b**) BIAS for $\hat{Q}=\gamma I$ corresponding to a scaled identity matrix. Please note the log values of γ for the x-axis. The red star corresponds to BIAS=0 and the intensity vector to case 1.

The results of varying β on BIAS and R_MSE_ are presented in Figure 4a. As mentioned previously, β simply act as a scaling factor, seen in Figure 1a. Because the models are scaled for the sake of comparison, its impact on the two quantities is similar for all three cases as long as α is kept fixed. The results are thus not presented graphically for the sake of shortness. From Equation (12), R_MSE_ is a ratio of trace so that the scaling parameter is eliminated. Thus, β has no impact and R_MSE_ will stay constant when β is varied. On the other hand, BIAS increases until the zero value is reached for the reference parameters. BIAS exceeds 0 for $\beta >\beta_{0}$. From β=3, the rate of change of BIAS with β decreases, i.e., degrading the assumed standard deviation of the range will only slightly impact the BIAS. A saturation to BIAS=0.8 occurs for β>4, which corresponds to a variation of 33% from the 0-reference value. As in 3.4.1., only values of BIAS>−1 are relevant, i.e., too overoptimistic values of β should be avoided, which corresponds in our case to β>1.2. Thus, the rate of change of BIAS for $\beta <\beta_{0}$ is much stronger than for $\beta >\beta_{0}$ as in the interval $\left[\begin{array}{cc} 1.2, & \beta_{0} \end{array}\right]$, BIAS varies more than 250%.

The combination of the results for BIAS from Figure 3b and Figure 4a highlights the possibility to counterbalance the effect of a higher α by decreasing β. Thus, different IM parameters lead to the same a posteriori variance factor. This result was also pointed out for a parametric correlation model [35] and is here related to the shape of $\sigma_{r}$, as shown in Figure 1.

#### 3.4.3. Note on the Scaled Identity Matrix, Case 1

For the sake of completeness, the impact of taking a scaled identity matrix to weight the observations, i.e., $\hat{Q}=\gamma I$, is presented in Figure 4b for case 1. The scaling parameter γ was varied from 500 to 10^7^ so that the 0-reference value for BIAS (red star) could be reached inside the range of values of γ. As mentioned previously, R_MSE_ is independent of the scaling parameter and equals 0.046 for all γ. The difference remains small (4%) wrt. the reference, but is synonymous with a slight model misspecification. As expected, it corresponds to the value found by taking α=0, and is thus coherent with the previous results. We further note that BIAS=0 for $\gamma_{0}=3850$. This optimal value is clearly related to the mean standard deviation of the range over all intensity values, i.e., 3780 mm. 

Thus, it is possible to use a scaled identity matrix as an alternative to a parametric VCM, which models the variance of range with an IM. This approximation remains correct as long as no strong variations of the Int occur, as mentioned in Section 3.4.1., and stays valid for cases where the variations of the range variance are stronger due to the low intensities values, as seen in case 2. Thus, there exists an optimal scaling of the identity matrix, which corresponds to the smallest ΔQ. This scaling parameter can be determined based on the value of the a posteriori variance factor wrt. to the a priori value. Similar results were obtained in Wujanz et al. [14]. This behaviour can be interpreted as due to the low level of heteroscedasticity of the observations.

### 3.5. Summary of the Simulations

From the results of the simulations, we propose to simplify the IM when used in a LS adjustment by taking a constant $\sigma_{r}$:

(14) $$\sigma_{r}=\beta_{0}{\left(\bar{I}nt\right)}^{\alpha_{0}}.$$

The validity of this approximation was shown to be limited to objects with homogeneous intensities, independently of low or high. With inhomogeneous intensities, we understand the presence of strong jumps in the intensity vector, as presented in case 3. When a constant variance is used in such cases, the LS adjustment, which estimates the CP of the B-splines approximation, is less efficient and the a posteriori variance factor biased, leading possibly to wrong interpretations of test statistics for deformation analysis. This is an undesirable effect. Consequently, to guarantee the efficiency of the LS adjustment, we propose to avoid this global approximation when the intensity vector is strongly varying. In this contribution, a range of variations of 100 Inc was identified as being allowed, which can be strengthen based e.g., on the results of the overall model test, provided that an accurate value of the a priori variance factor is used. If the intensities fluctuate, either a point-wise weighting [14] or a domain-wise weighting should be preferred. Independently of the underlying geometry of the object, the domains to which a constant variance are applied in the LS adjustment can be identified based on an analysis of the intensities. Timmen [20] proposed an innovative way to group the points of a point cloud using a quality index indicator adapted to each scanning configuration. However, it should be pointed out, that B-splines adjustments are more often used for simple objects, i.e., which could not be approximated by geometric primitives with a GHM. Consequently, strong variations will appear rarely.

The scaled intensity matrix is an alternative to the approximated VCM that accounts for heteroscedasticity with an intensity model, if the mean standard deviation of the range is known or can be approximated with the IM. This approximation holds true for domain-wise weighting.

In this contribution, we chose reference values for α and β. Our conclusions are not impacted by this choice. Variations would have led to a shift of the curve from Figure 3 or Figure 4, i.e., the minimum of BIAS and R_MSE_ would have been moved by the corresponding difference wrt. to the reference taken in this article.

## 4. Real Case Analysis

In the simulations, we chose intentionally to concentrate on the influence of varying the parameters of the approximated VCM for a simple geometry free case. We drew first conclusions in direction of a model simplification with constant variance. In this section, we wish to validate this model in a more general case, i.e., when a real curve is approximated. This corresponds thus to a less simple design matrix compared with the simulations with OMC observations.

### 4.1. Methodology

We use the original intensity vector presented in Figure 2, which corresponds to the case 1 of the simulations. A noise vector $l_{noise}$ is simulated, i.e., lnoise=Q0,MC12rdn, where rdn is a random vector of length n and $Q_{0,MC}$ the previously described reference VCM after mathematical correlations.

**Figure 5:**
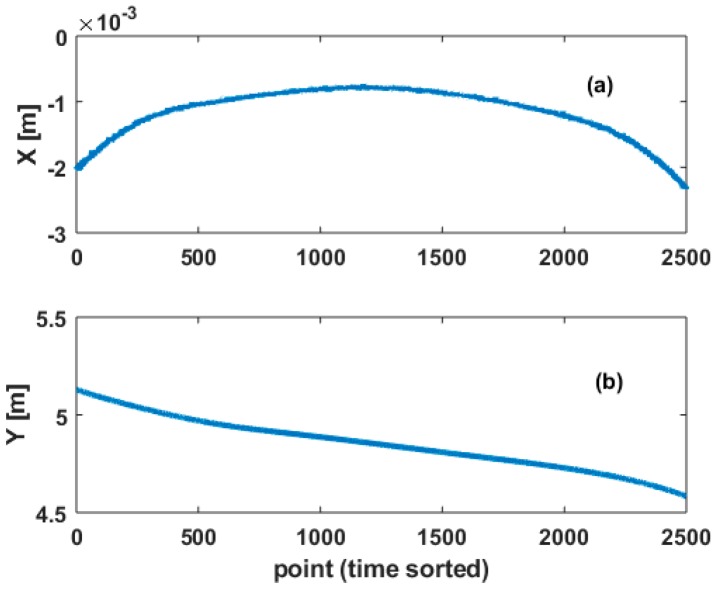
Simulated parametric B-splines curves. (**a**) X component; (**b**) Y component versus point number (i.e., time). To the geometrical observations is added a noise vector, with VCM $Q_{0}$. This real case study corresponds to an arch bridge with a known noise vector.

The generated noise vector is added to range and angle measurements obtained by a backward Cartesian to polar transformation of $l_{computed}$. This vector corresponds to a reference geometry, extracted from the original observations following the principle of Section 3.1. Figure 5 shows the final noisy observations transformed into Cartesian coordinate X and Y.

This reference curve is fitted using B-splines. Because of their smoothness properties [28], we use B-splines of order 3. 30 CP are estimated, following Zhao et al. [34]. The knot vector is determined by a Monte Carlo Method, which allows an optimal determination of the knots [27]. The columns of the design matrix used in Equation (1) depend on the B-splines and the knot vector, as seen in Equation (2).

**Figure 6:**
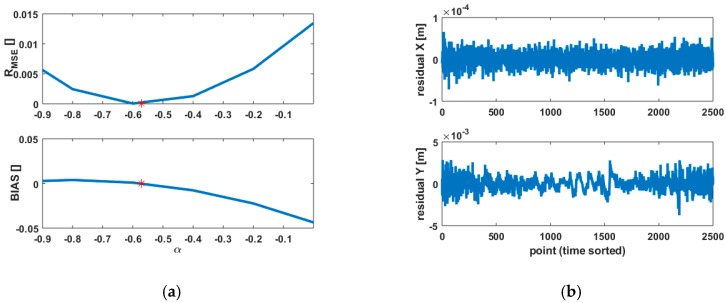
Results from a real case study with known noise vector. (**a**) R_MSE_ and BIAS of the a posteriori variance factor. The red star corresponds to the reference parameters $\alpha_{0}$; (**b**) residuals for the X (**up**) and Y (**bottom**) components. The Monte Carlo algorithm to compute the knot vector was used for a better accuracy of the B-splines approximation.

In a real case scenario, the stochastic properties of the LS residuals are unknown and can only be modeled. Moreover, the functional model is per essence imperfect due to suboptimal knot determination or CP number, i.e., the B-splines curve only approximate the observations. These misspecifications flow into the residuals in an unknown way as shown in Figure 6b, i.e., the discrepancy to a white noise vector is particularly visible for the Y-residuals.

Similarly to the simulations, the approximated VCM ${\hat{Q}}_{MC}$ used in the LS adjustment to determine the CP are computed by varying the parameters [α,β]. This strategy aims to investigate if a model simplification is possible without affecting the trustworthiness of the LS solution. As described in Section 2, mathematical correlations are taken into account using the law of variance covariance propagation resulting in a fully populated ${\hat{Q}}_{MC}$ with a block diagonal structure.

The theoretical quantities defined in Section 2 allow an interpretation of the impact of varying [α,β] without being influenced by the residuals. Nevertheless, Monte Carto simulations were additionally carried out by varying the random vector. As the results based on closed Equations (11) and (12) were confirmed, as seen in Figure 6a, they are not presented for the sake of shortness. The coordinates of the CP being similar for all cases due to the unbiasness of the LS estimator are not depicted.

### 4.2. Results

Figure 6a highlights that the impact of a more complicated design matrix on the quantities under consideration is negligible. Consequently, the conclusions previously drawn from the simulations stay valid for a more challenging scenario. It is moreover emphasized that the variations of BIAS around the 0 value are less strong than in the previous simulations, i.e., 0.005<BIAS<−0.05 for $\alpha \in \left[\begin{array}{cc} -0.9 & 0 \end{array}\right]$. Moreover, only $\alpha >\alpha_{0}$ leads to a slight underestimation of BIAS, which remains undetectable within the overall model test. The variations of R_MSE_ are four times smaller than in the simulations, highlighting that the loss of efficiency induced by using a scaled identity matrix (α=0) is negligible. Varying β in a range of values from 0.5 to 5 neither affected BIAS nor R_MSE_ significantly. Corresponding results are not presented here for the sake of shortness. As mentioned in Section 3, a good approximation of the standard deviation of the range by means of the IM remains however indispensable. As long as the intensity values are homogeneous, the factor of a scaled identity matrix can be alternatively deduced from the a posteriori variance factor.

Additionally, ${\hat{\sigma }}_{\hat{x}}^{2}$, i.e., the a posteriori variance of the residuals for the X component is computed [34] and compared to the known a priori value $\sigma_{0,\hat{X}}^{2}$ to validate the theoretical results. The ratio $R_{\hat{X}}=\frac{{\hat{\sigma }}_{\hat{X}}^{2}-\sigma_{0,\hat{X}}^{2}}{\sigma_{0,\hat{X}}^{2}}$ reaches −0.0068 for all α. Thus, a good adequation between the a priori and a posteriori variances is obtained for the **X** component, independently of the power factor chosen. Both the goodness of the knot vector, as well as the good approximation of $Q_{0}$ by means of $\hat{Q}$ are here highlighted.

## 5. Conclusions

A poorly estimated VCM affects the results of the LS adjustment, leading eventually to erroneous conclusions from test statistics. Therefore, a priori models of the variance of the measurements should be as accurate as possible, by staying at the same time simple and non-computational demanding. Based on physical considerations, a model to weight the range measurements of terrestrial TLS was recently proposed. This proposal makes use of the intensity values and shares the same concept as SNR models for e.g., GPS observations. Many factors influencing the precision of the range measurements such as the range itself, horizontal angle dependency, or reflectivities are contained and summarized in the intensity values. Based on empirical analysis using the 1D mode or residual analysis from LS adjustment, a power function was fitted to the empirical standard deviation of the range versus intensity. However, the procedure remains time consuming and necessitates a calibration of the instrument under consideration. Moreover, it is questionable if the power model parameters are usable in real scenarios, where other effects may affect the range variance. When TLS observations are post-processed for e.g., deformation analysis, the derived weighting model is applied in a generalized LS adjustment to compute the parameters of a plane with a GHM or the coordinates of the CP from a B-spline curve. In this contribution, we investigated the extent to which a simplification of the IM model is meaningful for such applications.

In real case scenarios, the exact VCM of the observations is unknown and assessing the influence of varying the parameters of a stochastic model is difficult if not impossible. However, simulations in a geometry free case allow studying the impact of stochastic model misspecifications. To that aim, three different intensity vectors were simulated. It led to the conclusion that taking a constant variance has a negligible impact on the trustworthiness of the LS solution, as long as the variations of the intensity values remain small. An interval of +/−100 Inc around the mean intensity value was proposed, which can be adapted depending e.g., on the results of the global test. The non-uniqueness of the parameter combination was further highlighted, i.e., the variations of the power factor had a similar affect as changing the scaling factor. This result was confirmed in a real case study, where geometry to estimate was more challenging. The computation of the constant variance relies on the mean of the intensity and makes use of the IM with calibrated parameters. The scanned object to be approximated with B-splines should be divided into domains of homogeneous intensities. This recommendation grandly simplifies the computation of the VCM of TLS measurements, as no point-wise weighting is necessary. Similarly, a parametric correlation model remains to be developed to account for temporal correlations of TLS measurements. This topic remains an open research field.
